# Mechanisms of breast cancer risk in shift workers: association of telomere shortening with the duration and intensity of night work

**DOI:** 10.1002/cam4.1135

**Published:** 2017-07-14

**Authors:** Johanna Samulin Erdem, Heidi Ødegaard Notø, Øivind Skare, Jenny‐Anne S. Lie, Marte Petersen‐Øverleir, Edyta Reszka, Beata Pepłońska, Shanbeh Zienolddiny

**Affiliations:** ^1^ Department of Chemical and Biological Work Environment National Institute of Occupational Health Oslo Norway; ^2^ Department of Occupational Medicine and Epidemiology National Institute of Occupational Health Oslo Norway; ^3^ Department of Toxicology and Carcinogenesis Nofer Institute of Occupational Medicine Lodz Poland; ^4^ Department of Environmental Epidemiology Nofer Institute of Occupational Medicine Lodz Poland

**Keywords:** Breast cancer, circadian, occupational, shift work, telomere length

## Abstract

Occupational factors such as shiftwork and especially night work that involves disruption of the circadian rhythm may contribute to increased breast cancer risk. Circadian disruption may also affect telomere length (TL). While short TL generally is associated with increased cancer risk, its association with breast cancer risk is inconclusive. We suggest that working schedules might be an important factor in assessment of effects of TL on breast cancer risk. Moreover, telomere shortening might be a potential mechanism for night work‐related breast cancer. In this study, effects of shift work on TL and its association with breast cancer risk were investigated in a nested breast cancer case–control study of Norwegian nurses. TL was assessed by qPCR in DNA from 563 breast cancer patients and 619 controls. Here, we demonstrate that TL is affected by intensive night work schedules, as work with six consecutive night for a period of more than 5 years was associated with decreased telomere lengths (−3.18, 95% CI: −6.46 to −0.58, *P* = 0.016). Furthermore, telomere shortening is associated with increased breast cancer risk in workers with long periods of consecutive night shifts. Thus, nurses with longer telomere lengths had a lower risk for breast cancer if they had worked more than four (OR: 0.37, 95% CI: 0.16–0.79, *P* = 0.014) or five (OR: 0.31, 95% CI: 0.10–0.83, *P* = 0.029) consecutive night shifts for a period of 5 years or more. These data suggest that telomere shortening is associated with the duration and intensity of night work and may be a contributing factor for breast cancer risk among female shift workers.

## Introduction

Breast cancer is the most common cancer in women worldwide. The etiology of the disease is complex and involves several known biological and lifestyle risk factors 1, 2. Hereditary genetic factors such as high‐risk mutations in breast cancer 1 and 2 (*BRCA1* and *BRCA2*) genes, as well as genetic polymorphisms in multiple genes including ATM serine/threonine kinase (*ATM*) and genes in the tumor protein 53/MDM2 proto‐oncogene pathway are also associated with increased risk 3, 4. Furthermore, occupational factors may contribute to increased risk of breast cancer. Several studies have shown an association between shift work and increased breast cancer risk in various occupational groups 5, 6, 7, 8, 9, 10. However, a recently published study did not confirm the suggested relationship between shift work and breast cancer, but rather, conclude that night shift work has little or no effect on breast cancer incidence 11.

The mechanisms for the association between night work and increased cancer risk are largely unknown. Work schedules including work at night have been shown to affect telomere length (TL) 12, 13, 14. TL varies among individuals and is also affected by several other factors including age, life style, health state, and environmental factors 15, 16, 17, 18, 19, 20. Telomeres, which consist of tandem (TTAGGG)_n_ nucleotide repeats, cap the ends of chromosomes and prevent chromosome shortening during replication. As telomeres are critically shortened, chromosome instability increases, and cellular senescence and apoptosis occur 21. Genomic instability following telomere shortening is widely accepted as a mechanism of tumor development 22. Telomere shortening is generally associated with increased cancer risk; however, data on TL and breast cancer risk are inconclusive. While some studies report telomere shortening in breast cancer 23, 24, others report increased breast cancer risk with longer telomeres 25, and yet other studies observe no association between TL and breast cancer risk 26, 27, 28. However, telomere shortening in breast cancer patients is correlated with severity of breast cancer stage and aggressiveness of breast cancer cell phenotype 24, 27, 29, 30. Moreover, an association between hereditary breast cancer and telomere shortening has been suggested 29, but has not been supported by other studies^31^.

Telomeres can be elongated by the nuclear enzyme complex telomerase which consists of telomerase reverse transcriptase (TERT), telomerase RNA component (TERC), and dyskerin 21. Polymorphisms in telomere maintenance genes can influence TL and cause dysregulation of telomere elongation leading to cell immortality, which is an important feature of cancer cells 32, 33. Accordingly, polymorphisms in telomere maintenance genes and upregulation of telomerase activity have been associated with breast cancer risk 34, 35, 36.

We have recently assessed night shift work by duration and intensity of night work and found that female nurses that had worked for more than 5 years in schedules with more than six consecutive nights had an increased breast cancer risk 6. It is, however, unclear if night work may contribute to reported variations in TL in breast cancer patients. In this study, we sought to investigate TL variation as a potential mechanism of the association between long duration of night shift with several consecutive nights and the increased risk of breast cancer. Furthermore, we also investigated whether changes in TL could be affected by functional genetic polymorphisms in the telomerase genes *TERT* and *TERC*.

## Material and Methods

### Study design and study population

**Figure 1:**
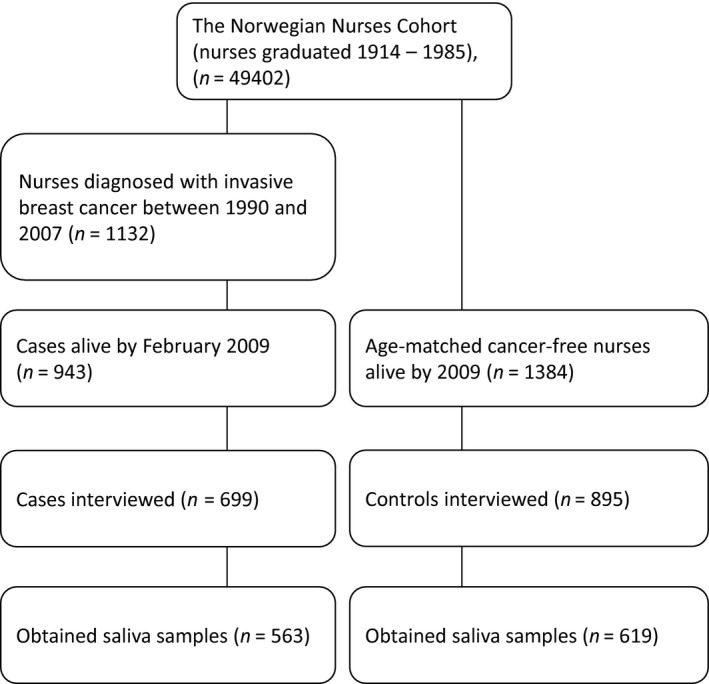
Flowchart illustrating the design of the nested case–control study.

This nested case–control study included Norwegian nurses graduated between 1914 and 1985. Study design, data collection, and recruitment of subjects were performed as previously described 6, and as outlined in Figure 1. Briefly, all cases were diagnosed with breast cancer between 1990 and 2007. Controls were frequency matched to cases by diagnosis year of the case and in 5‐year age groups. Only controls which were cancer‐free at and prior to the year of diagnosis of the case, were included. To be included in the study, cases and controls should be alive as of February 2009, had worked as a nurse for at least 1 year and had consented to be interviewed. Response rates were 74% among cases (699 women) and 65% among controls (895 women). In 2009, a few weeks prior to the telephone interviews, all cases and controls received an information letter containing a checklist for work history, a letter of consent, a request for saliva samples, and an Oragene saliva sampling kit (DNA Genotek Inc, Kanata, ON, Canada). During the telephone interview, information on potential breast cancer risk factors and lifetime occupational history was collected. Saliva samples were received from 563 cases and 619 controls. Both cases and controls gave full informed consent that their information could be used and published for research purposes, given that their personal details would remain anonymous. The study was approved by the Regional Committee for Medical and Health Research Ethics, South‐East region (S‐08430a, 2008/10453).

### Assessment of night work

Full details on the exposure assessment were described previously 6. Accordingly, night work included working periods from both rotating and permanent night schedules. Night shift was defined as a shift including work between 12 pm and 6 am. For each job, information on job duration, workplace, proportion of fulltime work, and work schedules (only day, only night, or both day and night shifts) was collected. Information on number of consecutive night shifts (intensity) was obtained from all jobs that included either permanent night work or rotating night shifts. Our analyses focused on the combination of duration and intensity of night work which is a more accurate exposure metric than just duration of night shifts, and which has been associated with increased risk of breast cancer 6. The following exposure metrics were used: “Duration of work including minimum *n* consecutive nights” (*n *=* *3–6), and four categories were defined. (1) never worked night shifts (reference group), (2) worked at night, however, never *n* consecutive night shifts, (3) worked *n* consecutive night shifts for <5 years, and (4) worked *n* consecutive night shifts for ≥5 years. Moreover, a metric focusing on the duration of night work only was used, and three categories were defined. (1) never night work (reference group), (2) 1–11 years of work including night shifts, and (3) ≥12 years of work including night shifts.

### DNA extraction and genotyping

DNA was extracted from the saliva samples using Oragene DNA isolation kit as described by the manufacturer (DNA Genotek Inc). Single nucleotide polymorphisms (SNPs) in *TERT* (rs2736108) or in the proximity of *TERC* (rs12696304 and rs10936599) were chosen based on previously published literature showing a connection between the selected SNPs and variations in TL 37, 38, 39, 40. Genotyping was performed by Taqman genotyping using 20 ng/*μ*L DNA on a 7900HT real‐time PCR system (Applied Biosystems, ThermoFisher Scientific, Waltham, MA) or by pyrosequencing using Pyromark Q24 Advanced technology (Qiagen, Hilden, Germany) according to the manufacturers’ instructions.

### Analysis of telomere length

Absolute TL was analyzed by qPCR using SYBR Green I technology essentially as previously described, with minor adjustments 41, 42. Accordingly, TL was analyzed using a relative standard curve approach. The multi copy gene ferritin heavy chain (*FTH1*) was used as reference gene. Primer sequences were as follows: Telomere forward primer 5′–CGGTTTGTTTGGGTTTGGGTTTGGGTTTGGGTTTGGGTT–3′, telomere reverse primer 5′–GGCTTGCCTTACCCTTACCCTTACCCTTACCCTTACCCT–3′, *FTH1* forward primer, 5′‐GATGATGTGGCTTTGAAGAACTTTGCCA‐3′, *FTH1* reverse primer, 5′‐CACCTCGTTGGTTCTGCAGCTTCATCA‐3′. Primer specificity was determined by melting point analysis. qPCR was performed using 1 ng template DNA in a total volume of 10 *μ*L containing PerfeCTa SYBR Green Fastmix, ROX (QuantaBioSciences, Gaithersburg, MD, USA). Cycling conditions were as follows: 95°C, 2 min followed by 40 cycles of 95°C, 10 sec and 60°C, 45 sec. The standard curve was generated by performing serial dilutions of plasmid DNA containing a 10mer oligonucleotide with TTAGGG repeats and one copy of *FTH1* sequence. pUC57 plasmid DNA (GenScript, Piscataway, NJ,USA) was added to each standard to maintain a constant amount of total DNA per reaction tube. Standard curves had *r* ^2^ > 0.975. A master standard dilution was made to ensure minimal variation between different runs.

### Statistical methods

#### Analysis of TL as outcome variable

TL as outcome variable was analyzed using a linear mixed model with a random intercept for plates to take into account the plate variation on measured TLs. The data were ln‐transformed prior to analysis to ensure a more homogeneous residual variation in TLs between plates. Separate analyses were performed for the following combinations of exposure variables: (1) cancer status (case vs. control), (2) night work exposure metrics (reference = only day shift), (3) interaction terms between cancer status and night work, and (4) SNP variables.

#### Analysis of breast cancer risk

The odds ratios of breast cancer were analyzed using logistic regression. TL (ln‐transformed), night work exposure metrics, interaction terms between TL and night work, and SNP variables were considered as exposure variables.

#### SNP genetic models

Four different genotype models were used to analyze the effect of SNPs on TL or on the odds ratio of getting cancer: free genotype model (reference = CC, CG, GG), recessive model (CC vs. CG/GG), dominant model (CC/CG vs. GG), and additive genotype model (0 = CC, 1 = CG, 2 = GG).

#### Adjustments for confounders

The list of potential confounders tested included: alcohol consumption, parity, mother's age at first birth, duration of daily occupational exposure to X‐rays, hormonal treatment last 2 years before diagnosis, and occurrence of familiar breast cancer. For analysis of cancer risk, adjustments for age at cancer diagnosis (case) or age at year of diagnosis of the corresponding case, that is, age at recruitment (control) were included. In the analysis of TL as outcome, additional adjustments were made for age at the saliva test and number of years since cancer diagnosis. This variable allows for a possible more rapid change in TL after cancer diagnosis, and partially corrects for possible bias induced by the time delay from cancer diagnosis to the saliva sample collection in TL analyses. All possible combinations of adjustment variables were compared and the combination that minimized the AIC criterion was chosen. Final adjustments were performed for relevant confounding factors as further detailed in the respective table footnotes.

### Statistical software

Statistical analyses were done using R (version 3.2.2). Linear mixed models and logistic regressions were analyzed, using the lme and glm functions, respectively. Characteristics of the study subjects were assessed by Chi‐square square (*chisq.test)* or Mann–Whitney *U*‐test (*wilcox.test)* as appropriate. *P* ≤ 0.05 was considered significant.

## Results

The demographics of cases and controls enrolled in the study are shown in Table 1, and further details on the recruited subjects have been previously described 6. The occurrence of familial breast cancer was significantly different between cases and controls (*P* < 0.001). As expected, some differences in the established risk factors, i.e., age, number of children, alcohol consumption, and hormone replacement therapy, were observed between cases and controls. However, these differences were not statistically significant.

**Table 1:** Characteristics of study subjects

Characteristic	Cases (*n *=* *563)	Controls (*n *=* *619)	*P*‐value
Age (years)^a^ **,** mean ± SD	54.47 (7.70)	54.48 (8.04)	0.74^b^
No. of children, mean ± SD	2.12 (1.17)	2.25 (1.28)	0.08^b^
Age at first birth (years), mean ± SD	26.85 (4.09)	26.74 (3.96)	0.70^b^
Breast cancer in first‐degree family^d^ (Y/N)	104/453	54/561	<0.001^c^
Alcohol consumption ≥twice/week (Y/N)	43/520	37/582	0.26^c^
Daily exposure to x‐rays (Y/N)	107/456	100/519	0.20^c^
Hormone therapy in the past 2 years^e^ (Y/N)	127/425	121/484	0.21^c^
Years from diagnosis^a^ to saliva sampling	8.01 (4.75)	8.14 (4.77)	0.61^b^

a Age at cancer diagnosis (case) or age at year of diagnosis of the corresponding case (control).

b Derived from Mann–Whitney *U*‐test (two‐sided).

c Derived from Pearsons Chi‐square test (two‐sided).

d Breast cancer in mother or sister.

e Hormone replacement therapy in postmenopausal women.

### Effects of night work on telomere length

TLs were not significantly different in nurses that had worked night shifts compared with those that had worked only days. Thus, duration of night work independent of the intensity of night shifts did not influence TL. However, working many consecutive night shifts for at least 5 years was correlated with reduced TL independent of case–control status (Table S1). While, the adjusted mean for TL was 30.42 for those working minimum three consecutive nights, it decreased to 26.72 for those working minimum six consecutive nights (Fig. S1). This effect was significant among nurses that had worked minimum six consecutive nights for at least 5 years (E: −3.18, 95% CI: −6.46 to −0.58, *P* = 0.016) independent of their case‐control status, but not in those that worked <5 years with intensive consecutive night shifts.

**Figure 2:**
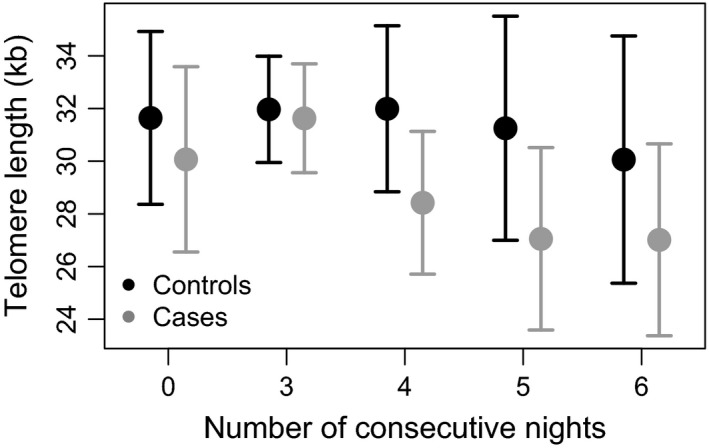
Changes in telomere lengths (kb) with increasing number of consecutive night shifts. Absolute telomere lengths were analyzed in DNA samples from cases and controls working 0, ≥3, ≥4, ≥5, and ≥6 consecutive night shifts for at least 5 years (mean ± SEM).

Similar patterns were observed for the differences in TL between cases and controls for the different measures of night work duration and intensity. For the different duration categories (no night work, <12 years, ≥12 years), no significant differences in TL were observed between cases and controls. However, the combined adverse effect of long duration and high intensity of night work on TL was generally stronger in cases than in controls (Figure 2). Among nurses with four and five consecutive nights for more than 5 years, TL was significantly shorter in cases than controls (E: −3.86, 95% CI: −7.57 to −1.01, *P* = 0.007, and E: −4.65, 95% CI: −9.49 to −0.96, *P* = 0.013), respectively (Table 2). A similar, however, not significant trend was observed for nurses that had worked more than six consecutive nights for more than 5 years (*P* = 0.075), based on 41 cases and 55 controls.

**Table 2:** Differences in telomere length between cases and controls in each category of the different night work schedules

Night work exposure	No. of Cases	No. of Controls	Difference in Telomere Length	CI	*P*‐value^a^
Independent of work schedules	607	554	−0.85	−2.31–0.41	0.187
Duration of work including night work					
Never night work	93	73	−1.39	−5.16–2.00	0.418
1–11 years	364	321	−0.59	−2.43–1.10	0.491
≥12 years	150	160	−1.07	−3.78–1.38	0.386
Duration of work including minimum 3 consecutive nights					
Never night work	93	73	−1.39	−5.14–2.00	0.417
Never worked 3 consecutive nights	90	94	−2.74	−6.48–0.39	0.088
Worked <5 years with ≥3 consecutive nights	173	153	−0.11	−2.62–2.40	0.928
Worked ≥5 years with ≥3 consecutive nights	251	234	−0.43	−2.56–1.61	0.669
Duration of work including minimum 4 consecutive nights					
Never night work	93	73	−1.40	−5.11–2.02	0.413
Never worked 4 consecutive nights	275	248	0.02	−2.01–2.04	0.983
Worked <5 years with ≥4 consecutive nights	136	123	0.47	−2.25–3.34	0.727
Worked ≥5 years with ≥4 consecutive nights	103	110	−3.86	−7.57 to –1.01	***0.007***
Duration of work including minimum 5 consecutive nights					
Never night work	93	73	−1.39	−5.08–1.99	0.416
Never worked 5 consecutive nights	343	315	0.17	−1.60–2.00	0.842
Worked <5 years with ≥5 consecutive nights	117	105	−0.99	−4.13–1.94	0.497
Worked ≥5 years with ≥5 consecutive nights	54	61	−4.65	−9.49 to –0.96	***0.013***
Duration of work including minimum 6 consecutive nights					
Never night work	93	73	−1.40	−5.09–1.99	0.413
Never worked 6 consecutive nights	371	337	0.08	−1.62–1.81	0.925
Worked <5 years with ≥6 consecutive nights	102	89	−1.57	−5.16–1.67	0.336
Worked ≥5 years with ≥6 consecutive nights	41	55	−3.58	−8.47–0.34	0.075

Separate analyses were done for each exposure metric. Adjustments were based on the AIC criterion. Here, models without adjustments were chosen.

a Derived from linear mixed model with a random intercept for plates. *P*‐values ≤ 0.05 were considered significant and are indicated in italics.

Finally, cases that had worked a minimum of five or six consecutive nights for at least 5 years had significantly shorter TL than cases working only day shifts (E: −3.73, 95% CI: −7.92 to −0.34, *P* = 0.030 and E: −3.88, 95% CI: −8.17 to −0.41, *P* = 0.028), (Table 3). No differences in TL were observed between these groups in the controls.

**Table 3:** Differences in telomere length between nurses working night work and those working only days

	No.	Difference in Telomere Length	CI	*P*‐value^a^
Cases				
Never night work	93	Reference group		
Worked ≥12 years night work	160	−0.15	−3.34–2.89	0.918
Worked ≥5 years with ≥3 consecutive nights	234	1.07	−1.86–4.14	0.464
Worked ≥5 years with ≥4 consecutive nights	110	−2.27	−5.89–0.77	0.142
Worked ≥5 years with ≥5 consecutive nights	61	−3.73	−7.92 to –0.34	***0.030***
Worked ≥5 years with ≥6 consecutive nights	55	−3.88	−8.17 to −0.41	***0.028***
Controls				
Never night work	73	Reference group		
Worked ≥12 years night work	150	−0.47	−2.64–2.73	0.748
Worked ≥5 years with ≥3 consecutive nights	251	0.11	−2.74–2.86	0.935
Worked ≥5 years with ≥4 consecutive nights	103	0.19	−3.10–3.48	0.905
Worked ≥5 years with ≥5 consecutive nights	54	−0.47	−4.33–3.40	0.803
Worked ≥5 years with ≥6 consecutive nights	41	−1.70	−5.95–2.34	0.399

a Derived from linear mixed model with a random intercept for plates. Adjustments were based on AIC criterion, and models without adjustments were chosen. *P*‐values ≤ 0.05 were considered significant and are indicated in italics.

### Effects of telomere length on cancer risk in nurses working night shifts

TL did not affect breast cancer risk when work schedules were not considered (OR: 0.80, 95% CI: 0.58–1.11; *P* = 0.177). Nor, when only the duration of night work, and not the intensity of work, was evaluated. However, longer TLs were associated with decreased odds for breast cancer in nurses that had worked a minimum of four and five consecutive nights for at least 5 years (OR: 0.37, 95% CI: 0.16–0.79; *P* = 0.014, and OR: 0.31, 95% CI: 0.10–0.83; *P* = 0.029, respectively), Table 4.

**Table 4:** Odds ratios (ORs) of developing breast cancer given a 1‐unit increase in telomere length (log scale). ORs were computed for each category of the night work exposure variables

Night work exposure	No. of Cases	No. of Controls	OR^1^	CI	*P*‐value^2^
Independent of work schedules^3^	607	554	0.80	0.58–1.11	0.177
Duration of work including night work^4^					
Never night work	91	71	0.75	0.29–1.88	0.536
1–11 years	357	318	0.83	0.54–1.28	0.399
≥12 years	145	156	0.78	0.42–1.42	0.414
Duration of work including minimum 3 consecutive nights^5^					
Never night work	91	71	0.75	0.29–1.88	0.534
Never worked 3 consecutive nights	87	92	0.52	0.22–1.15	0.117
Worked <5 years with ≥3 consecutive nights	169	153	0.98	0.53–1.82	0.952
Worked ≥5 years with ≥3 consecutive nights	246	229	0.85	0.51–1.40	0.517
Duration of work including minimum 4 consecutive nights^6^					
Never night work	91	71	0.75	0.29–1.88	0.533
Never worked 4 consecutive nights	266	244	0.98	0.60–1.60	0.924
Worked <5 years with ≥4 consecutive nights	133	123	1.10	0.55–2.22	0.788
Worked ≥5 years with ≥4 consecutive nights	103	107	0.37	0.16–0.79	***0.014***
Duration of work including minimum 5 consecutive nights^7^					
Never night work	91	71	0.73	0.28–1.85	0.505
Never worked 5 consecutive nights	332	308	1.04	0.66–1.62	0.880
Worked <5 years with ≥5 consecutive nights	116	105	0.77	0.37–1.59	0.477
Worked ≥5 years with ≥5 consecutive nights	54	61	0.31	0.10–0.83	***0.029***
Duration of work including minimum 6 consecutive nights^8^					
Never night work	91	71	0.73	0.28–1.85	0.506
Never worked 6 consecutive nights	359	330	1.00	0.66–1.54	0.985
Worked <5 years with ≥6 consecutive nights	102	89	0.67	0.28–1.54	0.344
Worked ≥5 years with ≥6 consecutive nights	41	55	0.42	0.13–1.14	0.110

^1^OR (odds ratio) was calculated on ln‐transformed telomere lengths. ^2^Derived from logistic regression and adjusted using the AIC criterion. *P*‐values ≤ 0.05 were considered significant and are indicated in italics. Separate analyses were done for each night work exposure variable. Adjusted for ^3,4,5,6^parity and occurrence of familiar breast cancer, and ^7,8^alcohol consumption and occurrence of familiar breast cancer.

### Associations of TL with TERT and TERC polymorphisms

We also examined the association between changes in TL and three polymorphisms in the *TERT* gene and in proximity of the *TERC* gene previously reported to affect the regulation of TL. Minor allele frequencies in cases and controls are shown in Table S2. Analysis using various genotype models showed that the previously reported SNPs (*TERT*: rs2736108, and *TERC*: rs12696304 and rs10936599) were not associated with changes in TL, nor was any correlation found to breast cancer risk in this cohort (data not shown).

## Discussion

TL is affected by several life style factors, and sleep deprivation and circadian disruption affect TL and telomerase activity 12, 13. Work schedules have been suggested to affect TL 14, but the effects of work including night work schedules have not been thoroughly investigated. Thus far, only one report addresses the effects of rotating night shifts on TL. Accordingly, Liang et al. reported no significant effects of night work on TL, but demonstrated a trend to shorter TLs in nurses with long history (>20 year) of rotating night shifts^12^. Our findings are in agreement with this previous study, showing a similar trend but no significant changes in TL when applying the crude exposure measure “duration of night work”, that is, years of rotating night shift work. In this study, we observed a trend of a decreased TL among women working minimum 5 years with several consecutive night shifts. This was significant among all nurses that had worked minimum six consecutive night shifts, when disregarding the case‐control status.

Shift work has been classified as a probable carcinogen and is suggested as a risk factor for breast cancer 43. Several studies have shown an association between shift work and increased breast cancer risk in various occupational groups 6, 7, 8, 9, 10, however, a recent comprehensive study on the relationship between shift work and breast cancer incidence concluded that night shift work, including long‐term shift work, has little or no effect on breast cancer incidence 11. The mechanisms behind breast cancer related to night work are also not well established. Our current findings, suggesting that the number of consecutive nights is important in telomere shortening, is interesting in light of previous published data demonstrating increased breast cancer risk only in nurses that had worked a minimum of six consecutive nights for at least 5 years 6. Thus, while telomere shortening contributes to tumor progression and is generally associated with increased cancer risk 44, 45, an association with breast cancer risk is inconclusive, as contradicting results on TL and breast cancer risk have been reported 23, 24, 25, 26, 27, 28. In line with several previous studies 26, 27, 28, we here observed no differences in TL in cases and controls and no association with breast cancer risk when disregarding night work. However, when evaluating the TL in cases and controls working night shifts, significantly shorter TLs were observed in cases than controls working four consecutive night shifts for more than 5 years. Moreover, this was associated with an increased risk for breast cancer. Interestingly, the association was not found when evaluating the combined effects of TL and the overall duration of years with night works when disregarding number of consecutive night shifts.

These data suggest that telomere shortening may contribute to increased breast cancer risk in workers that have worked many years with several consecutive night works (i.e., slow‐rotating shift systems). Such shift systems may cause disruptions of circadian rhythms and disruptions of sleep patterns 46, and thereby influence TLs which are regulated by core circadian genes 13. Genomic instability as a consequence of telomere shortening is a known mechanism in tumor development 22. The circadian clock regulates cellular responses to DNA damage, including several components of the DNA repair pathway, which maintain genetic stability and protect DNA integrity 47. Thus, night work involving circadian disruption may lead to telomere instability and dysregulation of DNA repair, which together may contribute to breast cancer among shift workers.

Telomerase activity oscillates with the circadian rhythm and is under control of *CLOCK* genes 13. Telomerase is responsible for maintaining the length of telomeres and disruption in the rhythmic telomerase activity gives shortened TL. Numerous polymorphisms in the genes encoding the two subunits of the protein (TERT and TERC) may cause dysfunction of telomere biology and be associated with cancer risk 25, 34, 35, 36, 37, 38, 39, 40, 48. We here investigated the effects of three previously reported functional polymorphisms in the *TERT* and *TERC* genes 37, 38, 39, 40 on TL and breast cancer risk. We observed no effects of the rs12696304, rs10936599, and rs2736108 SNPs on TL or breast cancer risk among nurses. This might indicate that night shift work affects TL independently of these genetic loci.

For evaluation of the effects of night work on cancer risk, the duration of night work is generally utilized. Previous studies evaluating the association of breast cancer and night work differ in respect to classification of duration of night work, with limits set between 3.1 and 30 years, making comparison of reports difficult as differences in shift systems may affect the results 49. The exposure metric of this study, including both duration and intensity of night work, is more accurate than metrics of several other papers concerning shift work and breast cancer 6. Moreover, this study is strengthened as only one profession was studied, thereby reducing problems with confounding factors of occupational exposure. However, in interpretation of our results it is important to consider the following limitations. Saliva is an easy accessible and noninvasive source of DNA, which may increase study participation rates. Most studies use blood samples for analysis of TL in case–control studies. Saliva samples consist of a mixture of different cell types including epithelial and white blood cells. However, available data suggest that there is a good correspondence between TL in different tissues of an individual 50. It should be noted that the time span between diagnosis and DNA sampling varies between the study subjects. The average time between diagnosis and sampling were not significantly different in cases and controls. To minimize impact of varying time spans on TL, additional adjustments for age at saliva sampling and number of years since diagnosis were included in the statistical analysis of TL as outcome. While southern blot analysis is the gold standard of relative TL measurement, qPCR methods give results in close correlation with Southern blot and are commonly used in analysis of absolute TLs 42. Finally, multiple procedures for determining confounders and covariate selection are available. In this study, the AIC criterion was chosen, as it is a commonly used method with the advantage that it allows for easy automatic comparison of all possible models. A criterion more focused on the precision of the exposure estimates, for example, Focused Information Criteria could also be considered. However, to our knowledge this method is not yet implemented in any statistical package.

In conclusion, this study demonstrates that telomere shortening is affected by work schedules and is correlated with long duration of work involving consecutive night shifts. Furthermore, reduced TL is associated with increased breast cancer risk in workers with long periods of consecutive night shifts. These data suggest that telomere shortening may be a contributing factor for breast cancer risk among workers with consecutive night work schedules.

## Conflict of Interest

None declared.

## Supporting information


**Figure S1.** Changes in telomere lengths (kb) with increasing number of consecutive night shifts. Absolute telomere lengths were analyzed in DNA samples from nurses working 0, ≥3, ≥4, ≥5, and ≥6 consecutive night shifts for at least 5 years.

**Table S1**. Difference in telomere length (TL) between night work schedules, independent of case‐control status.

**Table S2**. Genotype frequencies of *TERT* and *TERC* polymorphisms among breast cancer cases and control subjects.
