# Supplementary figures and images for: Mechanisms of breast cancer risk in shift workers: association of telomere shortening with the duration and intensity of night work
Source: Cancer Med. 2017 Jul 14;6(8):1988–97. doi: 10.1002/cam4.1135 (PMC5548875; doi:10.1002/cam4.1135)

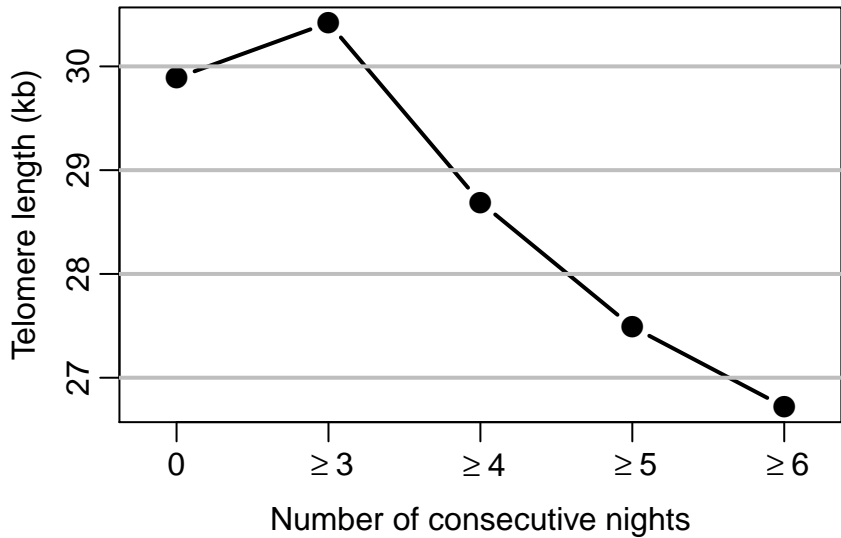

Supplement: Supplementary file 1 — Figure S1. Changes in telomere lengths (kb) with increasing number of consecutive night shifts. Absolute telomere lengths were analyzed in DNA samples from nurses working 0, ≥3, ≥4, ≥5, and ≥6 consecutive night shifts for at least 5 years. [file CAM4-6-1988-s001.pdf]
