# Supplementary material for: Mechanisms of breast cancer risk in shift workers: association of telomere shortening with the duration and intensity of night work
Source: Cancer Med. 2017 Jul 14;6(8):1988–97. doi: 10.1002/cam4.1135 (PMC5548875; doi:10.1002/cam4.1135)
Supplement: Supplementary file 2 — Table S1. Difference in telomere length (TL) between night work schedules, independent of case‐control status. [file CAM4-6-1988-s002.docx]

Supplementary Table 1. Difference in telomere length (TL) between night work schedules, independent of case/control status.

| Night work exposure | | No. | TL | Difference in TL | 2.5% | 97.5% | P-value |
| --- | --- | --- | --- | --- | --- | --- | --- |
| Duration of work including night work | | | | | | | |
| Never night work | | 166 | 29.90 | Reference group | | | |
| 1-11 years |  | 685 | 30.36 | 0.46 | -1.49 | 2.46 | 0.634 |
| ≥ 12 years |  | 310 | 29.49 | -0.41 | -2.65 | 1.69 | 0.694 |
| Duration of work including minimum 3 consecutive nights | | | | | | | |
| Never night work | | 166 | 29.89 | Reference group | | | |
| Never worked 3 consecutive nights | | 184 | 29.42 | -0.47 | -2.92 | 1.89 | 0.685 |
| Worked ˂ 5 years with ≥ 3 consecutive nights | | 326 | 29.96 | 0.06 | -2.11 | 2.21 | 0.952 |
| Worked ≥ 5 years with ≥ 3 consecutive nights | | 485 | 30.42 | 0.53 | -1.5 | 2.61 | 0.598 |
| Duration of work including minimum 4 consecutive nights | | | | | | | |
| Never night work | | 166 | 29.91 | Reference group | | | |
| Never worked 4 consecutive nights | | 523 | 30.78 | 0.87 | -1.1 | 3.01 | 0.380 |
| Worked ˂ 5 years with ≥ 4 consecutive nights | | 259 | 29.90 | -0.01 | -2.26 | 2.23 | 0.996 |
| Worked ≥ 5 years with ≥ 4 consecutive nights | | 213 | 28.69 | -1.21 | -3.67 | 0.98 | 0.274 |
| Duration of work including minimum 5 consecutive nights | | | | | | | |
| Never night work | | 166 | 29.91 | Reference group | | | |
| Never worked 5 consecutive nights | | 658 | 30.61 | 0.70 | -1.23 | 2.74 | 0.466 |
| Worked ˂ 5 years with ≥ 5 consecutive nights | | 222 | 30.03 | 0.13 | -2.19 | 2.44 | 0.909 |
| Worked ≥ 5 years with ≥ 5 consecutive nights | | 115 | 27.49 | -2.41 | -5.42 | 0.07 | 0.057 |
| Duration of work including minimum 6 consecutive nights | | | | | | | |
| Never night work | | 166 | 29.90 | Reference group | | | |
| Never worked 6 consecutive nights | | 708 | 30.29 | 0.39 | -1.55 | 2.35 | 0.684 |
| Worked ˂ 5 years with ≥ 6 consecutive nights | | 191 | 31.19 | 1.29 | -1.05 | 3.88 | 0.275 |
| Worked ≥ 5 years with ≥ 6 consecutive nights | | 96 | 26.72 | -3.18 | -6.46 | -0.58 | 0.016 |
