# Supplementary material for: Mechanisms of breast cancer risk in shift workers: association of telomere shortening with the duration and intensity of night work
Source: Cancer Med. 2017 Jul 14;6(8):1988–97. doi: 10.1002/cam4.1135 (PMC5548875; doi:10.1002/cam4.1135)
Supplement: Supplementary file 3 — Table S2. Genotype frequencies of TERT and TERC polymorphisms among breast cancer cases and control subjects. [file CAM4-6-1988-s003.docx]

Supplementary table 2. Genotype frequencies of *TERT* and *TERC* polymorphisms among breast cancer cases and control subjects.

| **Genotype** | **Cases** | **Controls** |
| --- | --- | --- |
| **rs2736108 C > T** |  |  |
| CC | 109 | 103 |
| CT | 82 | 86 |
| TT | 17 | 21 |
| MAF^a^: T | 0.279 | 0.305 |
| **rs12696304 C > G** |  |  |
| CC | 285 | 304 |
| CG | 210 | 231 |
| GG | 42 | 47 |
| MAF: G | 0.274 | 0.279 |
| **rs10936599 C > T** |  |  |
| CC | 252 | 251 |
| CT | 181 | 207 |
| TT | 41 | 50 |
| MAF: T | 0.278 | 0.302 |

^a^Minor allele frequency
